# An EEG-based mental workload estimator trained on working memory task can work well under simulated multi-attribute task

**DOI:** 10.3389/fnhum.2014.00703

**Published:** 2014-09-08

**Authors:** Yufeng Ke, Hongzhi Qi, Feng He, Shuang Liu, Xin Zhao, Peng Zhou, Lixin Zhang, Dong Ming

**Affiliations:** Laboratory of Neural Engineering and Rehabilitation, Department of Biomedical Engineering, College of Precision Instruments and Optoelectronics Engineering, Tianjin UniversityTianjin, China

**Keywords:** passive brain computer-interface, mental workload, EEG, feature selection, cross-task, working memory task, multi-attribute task

## Abstract

Mental workload (MW)-based adaptive system has been found to be an effective approach to enhance the performance of human-machine interaction and to avoid human error caused by overload. However, MW estimated from the spontaneously generated electroencephalogram (EEG) was found to be task-specific. In existing studies, EEG-based MW classifier can work well under the task used to train the classifier (within-task) but crash completely when used to classify MW of a task that is similar to but not included in the training data (cross-task). The possible causes have been considered to be the task-specific EEG patterns, the mismatched workload across tasks and the temporal effects. In this study, cross-task performance-based feature selection (FS) and regression model were tried to cope with these challenges, in order to make EEG-based MW estimator trained on working memory tasks work well under a complex simulated multi-attribute task (MAT). The results show that the performance of regression model trained on working memory task and tested on multi-attribute task with the feature subset picked-out were significantly improved (correlation coefficient (COR): 0.740 ± 0.147 and 0.598 ± 0.161 for FS data and validation data respectively) when compared to the performance in the same condition with all features (chance level). It can be inferred that there do exist some MW-related EEG features can be picked out and there are something in common between MW of a relatively simple task and a complex task. This study provides a promising approach to measure MW across tasks.

## Introduction

Contrary to active Brain-Computer Interfaces (aBCIs) using intentionally generated brain signals and allowing users to control devices through thoughts, passive BCIs (pBCIs) based on mental workload (MW) estimated from the spontaneously generated signals have shown potential in enhancing human-machine interaction performance, by driving machine adaptations when operators are found to be under high mental demand (Wilson and Russell, 2007; Coffey et al., 2010; Zander and Kothe, 2011). Cognitive monitoring which decodes real-time brain signals for operators’ ongoing cognitive states in human-machine systems has brought deep insights into the understanding of the brain at work, thereby inspiring researches on pBCIs. Such a pBCI could monitor workload online using electroencephalogram (EEG; Gevins et al., 1998; Berka et al., 2007; Brouwer et al., 2012), functional near infrared spectroscopy (fNIRS; Ayaz et al., 2010, 2012), electrocardiogram (ECG; Hoover et al., 2012; Ranganathan et al., 2012) and other psychophysiological measures and then reallocate tasks between the operator and the automation system when psychophysiological measures indicate high workload. Such a closed-loop system has been found to be capable of improving performance of human-machine systems and reducing operator’s subjective MW in some simulation tasks, such as an uninhabited air vehicle task (Wilson and Russell, 2007; Parasuraman et al., 2009), a human-robot cooperation task (Solovey et al., 2012), and a multi-attribute task (Prinzel et al., 2003).

To be precise, here used MW is a neuroergonomic concept and is mostly used in human factors studies. The theories of MW have long been debated, but there is no recognized and exact definition of MW so far. So exactly defining MW may be beyond the scope of this study. MW is generally considered to be correlated with task demand, time pressure, operator’s capacity, effort and performance (Paas et al., 1994). Instead of debating over definitions, many researchers believe that continuing to develop reliable MW measures will better promote progress in the application field. Therefore, the participants were usually instructed to devote their efforts to conduct tasks of different demands (difficulties) to induce different MW levels in existing MW studies.

Reliably estimating real-time MW is the first crucial step to build such a pBCI system. Because of its convenience and high temporal resolution, EEG has become the mostly studied MW indicator and has been found to be sensitive to MW changes in working memory tasks (Gevins et al., 1998; Pesonen et al., 2007; Lei and Roetting, 2011; Brouwer et al., 2012), simulated driving tasks (Lei and Roetting, 2011; Dijksterhuis et al., 2013), multi-attribute tasks (Laine et al., 2002; Prinzel et al., 2003; Christensen et al., 2012) and so on. In these studies, satisfactory results were mostly generated by classifiers trained and tested with EEG features from the same task. Some studies reported that alpha (8–12 Hz) power varied with changing MW due to its link to arousal level, idling and cortical inhibition (Pfurtscheller et al., 1996; Fink et al., 2005; Brouwer et al., 2012). Theta (4–7 Hz) power has been found to be modulated by mental efforts, and task requirements (Pesonen et al., 2007; Esposito et al., 2009). Delta, beta and even gamma bands have also been found to be associated with MW (Laine et al., 2002; Pesonen et al., 2007; Michels et al., 2010; Baldwin and Penaranda, 2012; Christensen et al., 2012). For example, Christensen et al. used EEG power and wave lengths of seven bands in the range of 0.5–100 Hz to classify MW in a multi-attribute task (Christensen et al., 2012). Classifiers used in MW studies include support vector machine (SVM; Brouwer et al., 2012; Christensen et al., 2012), artificial neural network (Gevins et al., 1998; Laine et al., 2002; Baldwin and Penaranda, 2012; Christensen et al., 2012), Bayes model (Wang et al., 2012), linear discriminant analysis (Christensen et al., 2012) and so on.

Although aforementioned studies on workload classification have indicated that spontaneous EEG is sensitive to MW changes and can serve as workload indices in online applications, the generalization (over time, across subjects and to new situations) of such classification-based decoding strategies remains to be an unsolved yet important question (Haynes and Rees, 2006). If a classifier trained with the data of a certain subject in a certain task at a certain time can only work well with the same subject in the same task at the same time, it would be inconvenient or even impossible for it to be applied to practical situations. In fact, the majority of existing studies are faced with problems just like this—classifiers were trained and tested on data from the same subject in the same task at the same time and thus good classification performances were reached. Until recent years, some researchers began to try to work on generalizable classifiers. Mühl et al. have successfully estimated workload across stressful and non-stressful affective contexts based on EEG features (Mühl et al., 2014). Three related studies have been reported in a special issue on Neuroergonomics in NeuroImage (Baldwin and Penaranda, 2012; Christensen et al., 2012; Parasuraman et al., 2012; Wang et al., 2012). Among these exploratory researches, cross-task MW estimation seems to be much more challenging. It was found that cross-task classification (classifiers were trained and tested on EEG features from different tasks) accuracies (average 44.8%, lower than chance level 50%) were significantly lower compared with within-task condition (average 87.1%) (Baldwin and Penaranda, 2012). These unsatisfactory results suggest that EEG-based cross-task MW classification remains to be a challenge. However, this problem is unavoidable for a practical design and further studies are required to be targeted to this challenge. Another recently published cross-task study attempted to cope with the challenge by training SVM on EEG features recorded from three relatively simple tasks (go/no-go, verbal n-back and reading span) (Walter et al., 2013). Each of the three tasks can induce nearly the same MW states and types of neural processing as in the two more complex learning tasks (working on diagram and algebra problems) used later to test the classifier (Walter et al., 2013). The cross-task classification accuracies, however, were not significant over chance level. The authors discussed that the poor performances may result from non-stationary patterns caused by the task order with advancing levels of difficulty, the use of different neural structures and executive functions due to the different nature of the tasks, and the varying absolute difficulty across the five tasks.

Based on existing cross-task studies, it can be summarized that the cross-task classification performance degradation may result from: (a) mismatched workload between training and testing data due to mismatched absolute task difficulty and/or subject’s different capacity among tasks (Baldwin and Penaranda, 2012); (b) the highly dissimilar EEG patterns invoked by different tasks because different tasks rely on different neural structures or types of cognitive process (Baldwin and Penaranda, 2012; Penaranda and Baldwin, 2012; Walter et al., 2013); and (c) the temporal effects and the non-stationary characteristics of EEG features resulting from irrelevant factors like circadian effects and fatigue (Baldwin and Penaranda, 2012; Walter et al., 2013).

There are also some cognitive neuroscience evidences supporting the necessity and feasibility to generalize MW estimator across various tasks. Cognitive resources theory, which believes that mental resources are limited, is one of the popular theories to interpret MW (Paas et al., 2003; Wickens, 2008). The overlapping aspects of MW of different cognitive functions may be that MW can be a measure of the amount of mental resources occupied by a task or the efforts one devotes to handle task demands (Wickens, 2008). From this point of view, MW may also be a measure of activation levels of the neural networks involved in a task or of the whole brain. The differences may be that different cognitive functions need different neural structures, adopt different processing strategies, and produce different neural responses and signals. Due to task-specific activations in various neural structures, EEG patterns generated under a certain task differs from those under another task, which makes it a challenge to recognize work load levels using only one general classifier. Generalizability is an essential property for a MW recognition model to be applied to real-world situations. But there is one thing in common that increasing task difficulty or demand may cause increasing in activation levels in the involved neural structures (Jonides et al., 1997; Carpenter et al., 1999; Meyer et al., 2000; Rietschel et al., 2012). The activation level changes of different neural structures may generate similar or same changes of EEG features, e.g., the changes in power spectrum and EEG coherence (Rietschel et al., 2012). As has been found that particular working memory task may evoke specific activations, however, various working memory tasks may also evoke similar difficulty-dependent activations in particular network (Langer et al., 2013). These underlying similarities can make it possible to create a generalizable classifier capable of handling WM recognition across various tasks at the same time. Therefore, efforts should and could be devoted to generalizing MW recognition model for real-world applications.

Existing studies attempted to classify working memory load across different working memory tasks (Baldwin and Penaranda, 2012) and to classify workload across relative simple tasks and complex learning tasks (Walter et al., 2013). In these cross-task studies, MW should be defined as a kind of physiological and psychological effect induced by task difficulty. Different task types or difficulty levels may result in different kinds of MW or different MW levels. In current study, we tried to cope with the possible factors that degrade the cross-task classification performance and thus find a way to make an EEG-based MW estimator trained on working memory tasks work well under the multi-attribute task. In other words, efforts were devoted to building an EEG-based MW recognition model which can be generalized across two different kinds of MW, namely working memory load and the more complex MW induced by a multi-attribute task. In terms of workload mismatching, it is hard to match the absolute task difficulty and a subject’s capacity between a memory task and a multi-attribute task. Even if task difficulty and subject’s capacities could be well-matched in laboratory settings by careful task design and satisfactory classification accuracy could be obtained, such a classifier would be difficult to be used in practical settings because of the uncontrollable task difficulty under actual working conditions. So, a regression model concerning more on COR and allowing error were used, instead of a classifier requiring precise equality in performance criteria. A more problematic issue may lie in the different EEG patterns evoked by different tasks. But no matter what the neural differences between tasks are, there should be similar EEG patterns which are sensitive to the changes of workload. The problem is how to pick out the MW-related EEG patterns from other-related ones according to supposition that some EEG features are related to MW and other features to other factors, such as task-types and temporal effects. Therefore, a specifically designed cross-task feature selection (FS) was examined to pick out the MW-related feature subset.

## Materials and methods

### Experimental design

Seventeen healthy on-campus college students (4 females and 13 males), ranging in age from 19 to 24, participated in the working memory tasks (verbal n-back and spatial n-back) and the Multi-Attribute Task Battery (MATB; Santiago-Espada et al., 2011), with written informed consent. Only one of them is left-handed. All the participants reported normal or corrected-to-normal visual acuity and no history of neurological or psychiatric illness. Their working memory performances were excellent in n-back task (accuracy above 90% in 2-back). The study was approved by the local ethics committee.

Firstly, all the participants were asked to complete 9 randomly presented 3.5-min blocks of task, including 4 blocks of verbal n-back task (*n* = 0, 1, 2, 3), 4 blocks of spatial n-back task and 1 block of resting task. The verbal n-back task used here was similar to the task used by Brouwer et al. (2012). The participants were asked to remember and compare each new letter to the letter presented *n* trials before it. The spatial n-back task was similar to the task used by Baldwin and Penaranda (2012). Participants were asked to remember and compare the current location of a white square to that occurred *n* trials before (25 alternative locations in total, 5 rows × 5 columns). During the inter-block interval (usually 2–4 min), participants took a rest until they felt able to go on with the task. Each block consisted of 60 3.5-s trials (20 targets) except the resting task. In each trial, stimuli successively appeared on a monitor for 0.5 s. For each stimulus, participants were instructed to press a certain button during the interval (3.5 s) from the onset of the current stimulus to the next onset indicating whether the current stimulus was a target or a non-target. Trials not responded during the response time-window were counted as being incorrect.

Once all nine blocks of task were completed, participants were instructed to perform the MATB task after a short rest. Four blocks of tasks (low, medium, high and a varying difficulty block) were designed with different overall difficulty levels manipulated by varying the demands of each subtask.. The data of the varying difficulty block was not used in current study. The four blocks of tasks were presented to the participants in a random order, and the participants were not informed of the difficulty level of their ongoing task. Each block of the low, medium and high difficulty tasks lasted 5.5 min. At the end of each block, participants were asked to rank a multidimensional subjective workload assessment—NASA Task Load Index (TLX; Santiago-Espada et al., 2011).

In an attempt to reduce learning effects, participants were asked to keep practicing until performance scores reached asymptote with minimal errors. The tasks used in practice were specifically designed and different from the tasks used in the experiment.

### Data acquisition and processing

EEG acquisition was performed in a magnetically and electrically shielded room. 30-channel EEG data (FP1, FP2, F7, F3, FZ, F4, F8, FT7, FC3, FCZ, FC4, FT8, T3, C3, CZ, C4, T4, TP7, CP3, CPZ, CP4, TP8, T5, P3, PZ, P4, T6, O1, OZ, O2) was recorded with ground at FPz using a NeuroScan NuAmps 40-channel amplifier. Impedances for all EEG channels were below 5 kΩ and were mentioned with an impedance meter prior to data collection. EEG data was sampled at 1000 Hz and high-pass filtered with cutoff frequency of 0.1 Hz.

All the signal processing procedures—preprocessing, feature extraction and model building—were performed using MATLAB. Data epochs contaminated by apparent artifacts (amplitude > 80 μV) due to eye blinks, significant muscle activity, and movements were rejected manually. EEG data recorded at each difficulty level of each task were then segmented into 2-s, non-overlapping segments and put into a power spectral density (PSD) estimator using Burg’s method (the order of an autoregressive model was 40, nfft = 1000). The sums of PSD in 7 frequency bands (δ: 0.5–3 Hz, θ: 3–8 Hz, α: 8–13 Hz, β1: 13–20 Hz, β2: 20–30 Hz, γ1: 30–45 Hz, γ2: 55–100 Hz) were extracted, resulting in 7 features of each of the 30 channels. The frequency band 45–55 Hz was not used due to the power line hum. Four input sets were created for each subject: verbal n-back inputs only (“V”); spatial n-back inputs only (“S”); verbal and spatial n-back inputs combined (“N”); MATB inputs only (“M”).

### SVM Regressor (SVMR) and cross-task estimation performance-based Recursive Feature Elimination (RFE)

Because the absolute workload level may not be matched between the working memory tasks and the MATB task and the relation between them was also unclear, any classifier that requires strict equality may generate a poor performance. Therefore, the regression algorithm SVMR, as implemented in LibSVM (Chang and Lin, 2011) with a linear kernel function, was used here. SVMRs were trained and tested on six different combinations of V, S, N and M. Each combination of training input set and testing input set was labeled accordingly. In within-task condition, there were four train-test combinations: VV, SS, NN and MM. For cross-task condition, there were two train-test combinations: NM and MN. For example, “VV” indicates the condition that both the training set and testing set came from verbal n-back task (V), and “NM” indicates the condition that the training set came from the verbal and spatial n-back inputs combined set (N) while the testing set came from the MATB inputs only (M). The four difficulty levels for both verbal and spatial n-back, 0-, 1-, 2- and 3-back, were labeled 1, 2, 3 and 4 respectively in training and testing SVMR (as the same to train a SVM classifier) due to the unavailability of the continuously changing workload. For the MATB task, the low, medium and high difficulty levels were labeled 1, 2 and 3 respectively.

**Figure 1:**
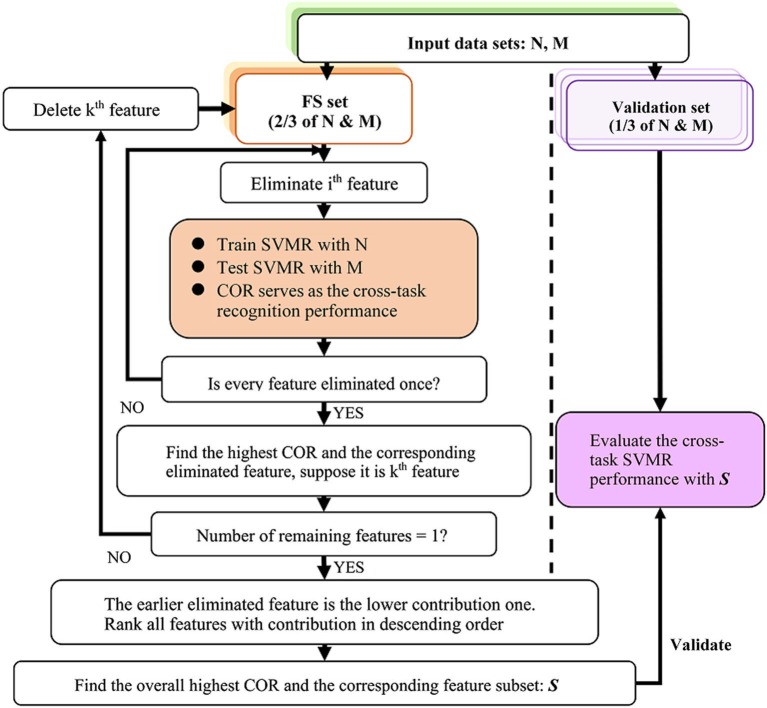
**The flow chart of the cross-task RFE algorithm**.

The Recursive Feature Elimination (RFE) algorithm has been successfully used in gene selection (Guyon et al., 2002). Here, the difference is that cross-task performance-based RFE, as shown in Figure 1, was based on the NM cross-task estimation performance, i.e., the COR between the predicted values and task difficulty of the test set. The feature subset picked out by this algorithm was regarded as the most salient feature set under NM condition. In order to avoid the possibility of circular inference, the data sets N and M were both divided into three parts and a 3-fold cross-validation was performed to separate the FS set and the validation set. In a fold, two parts were used as the FS set to get the salient feature subset (SF) and another part was used as the validation set to validate the feature subset. In order to relieve the effect of autocorrelation, the subsampling method used in cross-validation was not random sampling but sampling the data by time. The mean value of the three performances from the 3-fold cross-validation served as the final performance in statistical analysis.

## Results

### Behavioral data

For the verbal and spatial n-back task, repeated-measure ANOVAs were performed both on response time and accuracy. Main effects of task difficulty on response time and accuracy were found in both verbal and spatial n-back task (*p* < 0.01). Response accuracy decreased with increasing memory load with averages of 0.996, 0.991, 0.991 and 0.947 respectively for verbal n-back and 0.994, 0.984, 0.985 and 0.949 respectively for spatial n-back. Response time (ms) increased with the increasing memory load with averages of 490.046, 562.748, 721.225 and 780.512 respectively for verbal n-back and 511.847, 583.210, 699.854 and 799.437 respectively for spatial n-back.

In terms of the MATB task, repeated-measure ANOVAs were performed on the NASA-TLX measure. Main effect of task difficulty on TLX was found (*p* < 0.01), and the measure increased with the increasing task difficulty with averages of 42.353, 49.206 and 58.755, respectively.

### SVM regression results and the effects of cross-task RFE on within-task and cross-task conditions

**Figure 2:**
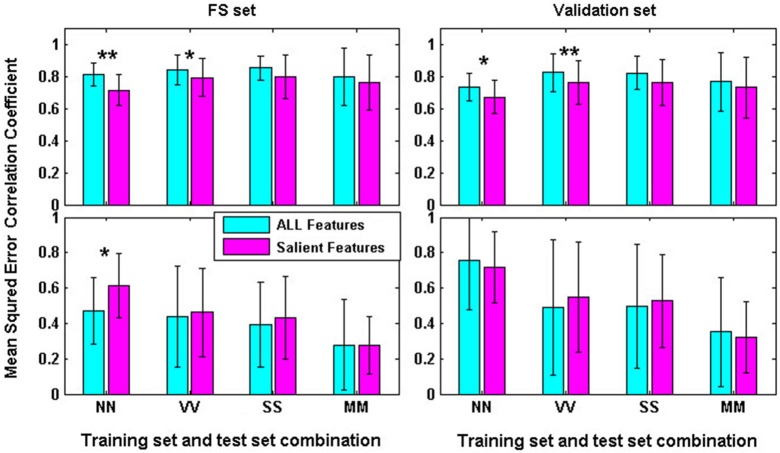
Within-task regression performances for FS and Validation data with all features and the salient features picked out by RFE (*: *p* < 0.05; **: ***p* < 0.01)**.

We firstly compared the within-task performances with all features (AF) and with the picked-out SF for both the FS set and the validation set respectively. As shown in Figure 2, the results show that both the COR and mean-squared error (MSE) were excellent under all conditions, though significantly declined CORs were found under NN and VV conditions for FS and validation data with SF compared to AF. For FS data, mean CORs were above 0.718 and mean MSEs were below 0.614. For validation data, mean CORs were above 0.675 and mean MSEs were below 0.755. Significant differences of both CORs and MSEs were also found between FS and validation data under all conditions (*p* < 0.01) except the VV-AF condition (*p* > 0.05) through paired *T-tests*. These results suggest that within-task regression performances were pretty good and the SF picked out by NM performance-based RFE only had minor effects on within-task performances.

**Figure 3:**
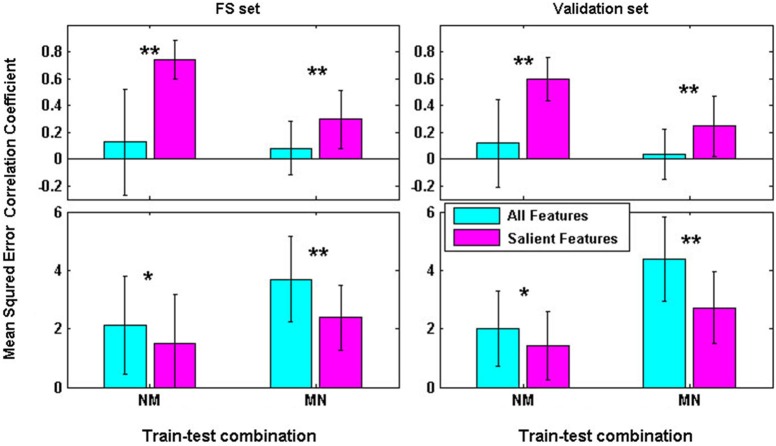
Cross-task performances for both FS and validation data with AF and SF respectively (*: *p* < 0.05; **: ***p* < 0.01)**.

Cross-task regression performances (as shown in Figure 3) with AF, however, were very disappointing. Under both NM and MN conditions, *T-test* results showed that CORs of cross-task regression with AF were not significantly different from zero although some satisfactory results were found for some subjects. Encouragingly, paired *T-tests* results showed that CORs were significantly increased for both FS and validation data with SF compared to AF under all the cross-task conditions (*p* < 0.01). MSEs were significantly reduced for both FS and validation data with SF under all conditions (*p* < 0.05). With the SF picked out by NM cross-task regression performance-based RFE, CORs and MSEs of FS and validation data under NM stood out from other conditions. Paired *T-tests* also indicated that CORs of FS data with SF were significantly greater than validation data with SF (*p* < 0.05). These surprising results suggest that the feature subset picked out by NM performance based RFE can significantly improve the cross-task regression performances, especially the NM performance. More satisfyingly, the within-task performances kept surprising. It can be inferred that the SF should be more workload-related and task-independent.

It can be definitely found that, the MSEs under cross-task conditions (mean above 1.488 and 1.422 for FS and validation respectively) were much larger compared to within-task conditions (mean value below 0.614 and 0.755 for FS and validation respectively). This kind of effect may result from the mismatched workload between training and testing data. Another reason may be the incapability of the labels used in training and testing to reflect the relative relationship between levels of workload in different tasks. In fact, the cross-task predicted values are bound to be off from the labels that can only reflect difficulty levels within a task. Therefore, only reliable is the relative relationship between the predicted values and the labels in cross-task conditions. That is exactly the reason why SVMRs, but not SVM classifiers, were used and why the CORs, but not the MSEs, served as the cross-task performance indicator.

**Figure 4:**
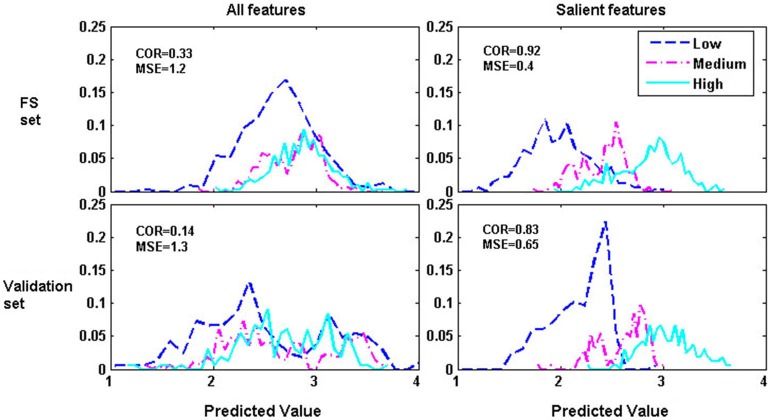
**Illustrations of the distribution curves of model predicted values for FS and validation data with AF and SF respectively under NM cross-task condition for one subject**.

Figure 4 illustrates the distribution curves of model predicted values under NM condition for one subject. It can be definitely found that the performances of both the FS and validation data were dramatically improved with SF compared to AF. The distribution of predicted values were higgledy-piggledy with AF but changed to be linear with the task difficulty with SF. These surprising results indicate that cross-task FS can indeed improve workload estimator’s generalization across tasks.

### The features picked out by cross-task RFE

**Figure 5:**
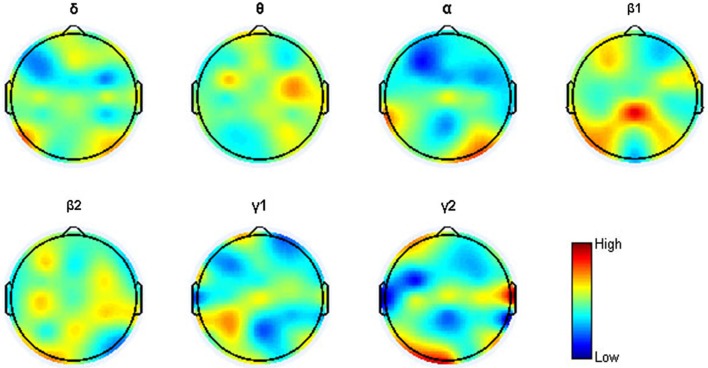
**Topographic mapping of the quantified feature contributions averaged across 17 subjects for the 7 frequency bands**.

As has been mentioned, the cross-task RFE algorithm can pick out the most SF and rank all the features according with their contributions to the NM cross-task SVMR performance. Then, questions such as how many and what kind of features were selected and what features contributed more in cross-task workload estimation may deserve more attention. It was found that the maximum performances were generally reached at smaller-sized feature subset at both the individual level and the average level. The average number of the salient features was 33 (2–71). It indicates that only a few features are more MW-related. Figure 5 indicates the topological distribution of each feature’s contribution to the NM cross-task performance. It can be found that the high contribution features distributed in a wide frequency and spatial range, and however, the features from frontal, parietal and occipital lobes showed higher contribution. The widespread high-contribution features may result from the fact that multiple cognitive processing and neural structures are involved when performing the tasks, especially the MATB task. Influenced by electromyogram, some high contribution locations can also be found at lateral sites, especially at the γ2 band.

## Discussion

The present study tested the effects of FS and regression model on the performance of EEG-based workload estimation across working memory task and MATB task. The results show that the salient features picked-out by NM performance-based cross-task RFE have significantly improved the MW estimation performance across working memory task and MATB task for both the FS and validation data, especially under NM condition. Compared with the existing two cross-task studies that have found the challenges in cross-task MW estimation (Baldwin and Penaranda, 2012; Walter et al., 2013), targeted methods were used to cope with the possible causes of the challenges. A regression model, instead of a classifier, was used to overcome the effect of possibly mismatched workload across the training and testing data. Based on the assumption that some EEG features are more related to the task nature and others are more sensitive to the changes of workload, a specifically-designed cross-task RFE was used to pick out the MW-sensitive features. The surprising results suggest that these methods indeed worked well and the possible causes been put forward should be reasonable. The major contribution of current study is that a viable approach has been found to improve the generalization of MW estimator across completely different tasks.

It should be noted that the tasks used in this study, visual verbal and spatial n-back and MATB, are very different from each other and rely on different neural structures or types of cognitive processing. Specifically, n-back tasks mainly rely on verbal or spatial memory and visual processing. Separately, verbal n-back also needs verbal processing, while spatial n-back needs spatial processing. But MATB is a set of different tasks, including system monitoring task, tracking task, communication task and resource management task (Santiago-Espada et al., 2011). Therefore, the MATB task may depend on a subject’s visuomotor processing, auditory perception, attention and so on. Different cognitive resource occupations or cognitive processing strategies may generate different EEG patterns. These differences of course will be the main impediment in estimating MW across tasks if not eliminated. In existing cross-task studies, MW estimation across three working memory tasks (Baldwin and Penaranda, 2012) and that across relatively simple tasks and complex learning tasks (Walter et al., 2013), task-specific patterns were not separated from the features used in MW estimation. That should be the major causes which led to the classification performance deterioration, except for the effect of mismatched MW. In the current study, the significantly improved cross-task performances by performance-based cross-task RFE suggests that the mechanism of MW and MW-related EEG patterns of different tasks should be, at least partially or in some aspects, identical. That is what make the generalization of MW estimation across tasks possible.

However, FS seemed to be not enough. On the one hand, the use of FS is based on the assumption that MW-related EEG patterns are separated from task-specific ones. But, the fact is not so simple. An EEG feature may be affected by MW and task nature at the same time. Therefore, other data mining methods should be examined in future studies. On the other hand, temporal effects has been found to be a strong interference in both within- and cross-task MW estimation (Baldwin and Penaranda, 2012; Christensen et al., 2012; Walter et al., 2013). However, the current methods may be incapable of coping with this challenge. These inadequacies may be just the reasons why the cross-task estimation performance has been greatly improved but is still incomparable to performance under within-task conditions.

One other issue of concern for cross-task performance is the large MSEs. It can be found that MSEs were much larger in cross-task (mean value above 1.400) than in within-task (nearly all below 1.000). One of the possible causes is that the labels used to train and test the model could only reflect the relative levels of difficulty within a task but not the absolute difficulty levels across tasks. Therefore, the mismatched workload would result in large errors if the predicted values were compared with the labels in cross-task condition. Furthermore, difficulty levels can easily remain stable within a block of n-back tasks but not so in complex simulated tasks, such as the MATB task. What’s more, workload is hard to be manipulated to maintain at a certain level, because workload depends on not only task difficulty but also efforts of the operator. We can only manipulate task difficulty through well-designs, but the mental effort is determined mainly by the operator. Possibly, operator’s efforts can fluctuate in the course of a task where the difficulty level remains stable. Therefore, MW should fluctuate rather than keep stable at a certain level in a block of a task. In fact, real-time changing workload is unavailable through current technologies and has not been considered in all the MW classification studies. These reflections above has motivated us to try a COR-guided regression model instead of a classifier.

It would be specially mentioned that this paper aims not to prove that verbal n-back, spatial n-back, and MATB tasks would cause the same activations or EEG patterns. Instead, it aims to look for feasibilities to generalize MW estimator across these tasks. Brain activations associated with different cognitive functions are very different. But this does not imply that there are nothing in common between workload induced by different tasks. It is worthwhile to find and give some evidence to this kind of possibility that the brain can generate similar EEG patterns when dealing with different difficulty levels presented by every different tasks. In addition, real-world tasks are much more complex, so a working memory task cannot comprise only working memory functions but some other functions as well, such as attention, visual or auditory processing, verbal or spatial processing, which are also needed to work together, just like coping with a more complex multi-attribute task. As an ergonomic concept, the term MW should concern more on the global “busy-state” of the brain rather than a specific kind of cognitive function.

It should be pointed out that the salient features picked out by RFE under NM condition has not been tested with novel tasks that did not participate in the FS process. Future studies should be committed to improving the generalization of features and MW estimation model to be task-independent. The methods used in this study should be tested on more different tasks. Also, other physiological measures like fNIRS (Ayaz et al., 2012) and ECG (Hoover et al., 2012), which have been found to be sensitive to MW but not been tested in cross-task MW estimation, should be an emphasis in future studies. In addition, this kind of cross-recognition has been put forward in other mental state studies, such as EEG-based emotional state estimation. The majority of studies on emotional state corresponding to specific emotional stimuli share an imperfection that EEG may be modulated by stimulus properties irrelevant to emotions (Bekkedal et al., 2011; Kim et al., 2013). In order for a computational model to be applied in the real word, it should be capable of predicting emotional states with various stimuli: picture stimuli, video stimuli, audio stimuli and so on (Kim et al., 2013). Other studies, such as mental fatigue and vigilance which are implemented under tasks and stimuli, may also encounter this kind of challenge.

## Conclusion

The current study was committed to meet the challenge of MW estimation across working memory tasks and simulated multi-attribute tasks through FS and a regression model. The results showed that the performances of regression-based cross-task MW estimation were greatly improved to a surprising level by using the feature subset picked out by RFE, compared to the estimation performance when using all features. It suggests that MW estimator trained on simple tasks is capable of estimating MW of a complex task through better selections of features and an improved modeling method. This study provides a promising approach to measure MW across tasks and even to build a task-independent model.

## Conflict of interest statement

The authors declare that the research was conducted in the absence of any commercial or financial relationships that could be construed as a potential conflict of interest.
